# Synthesis of (–)-Indolizidine 167B based on domino hydroformylation/cyclization reactions

**DOI:** 10.1186/1860-5397-4-2

**Published:** 2008-01-15

**Authors:** Giuditta Guazzelli, Raffaello Lazzaroni, Roberta Settambolo

**Affiliations:** 1 University of Manchester, School of Chemistry, Brunswick Street M13 9PL Manchester, UK; 2 Dipartimento di Chimica e Chimica Industriale, Università di Pisa, Via Risorgimento 35, 56126 Pisa, Italy; 3 ICCOM-CNR, Sezione di Pisa, Dipartimento di Chimica e Chimica Industriale, Università di Pisa, Via Risorgimento 35, 56126 Pisa, Italy

## Abstract

The synthesis of (–)-Indolizidine 167B has been achieved from optically active (*R*)-3-(pyrrol-1-yl)hex-1-ene. The key step is a highly regioselective hydroformylation reaction and a one-pot intramolecular cyclization providing a general approach to the indolizine nucleus.

## Background

**Figure 1:**
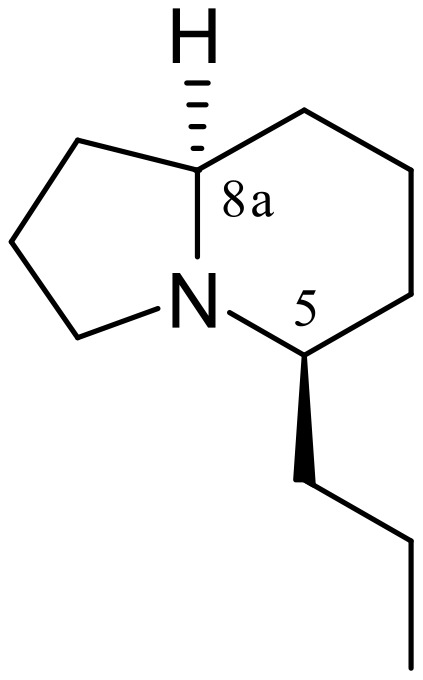
(–)-Indolizidine 167B.

Indolizidine alkaloids are widely diffused in nature and have attracted considerable attention because of their varied range of pharmaceutical application. Indolizidine 167B (Figure 1), one of the simplest amphibian indolizidine alkaloids, was originally identified as (*5R,9R*)-octahydroindolizine from the skin secretions of a frog belonging to the genus Dendrobates [1–2], which acts as noncompetitive blocker of neuromuscular transmission. Although the structure has been questioned [3], this alkaloid remains a target compound for many research groups [4–6].

**Scheme 1:**
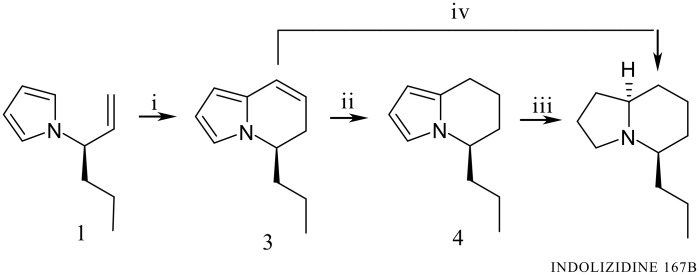
Reagents: i Rh_4_(CO)_12_, 30 atm CO:H_2_ = 1:1, 125 °C, toluene, 24 min, 76% yield; ii the same conditions as i 12 h under H_2_ 50 atm, after CO and H_2_ removal, 80% yield; iii 10 atm H_2_, Rh/C (5%), r.t. 45 min, 75% yield; iv H_2_ 10 atm, Rh/C (5%), r.t., 60 min, 64% yield.

**Scheme 2:**
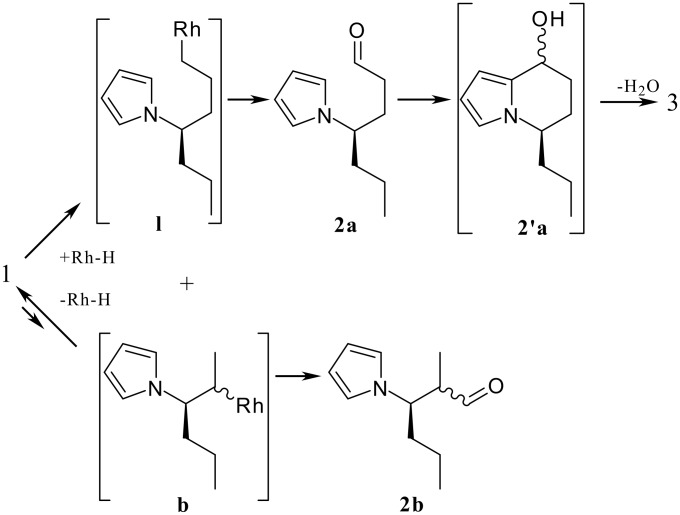
Stereospecific interconversion of the rhodium-alkyl intermediates as the key for regioselective formation of the linear aldehyde **2a.**

We recently reported the synthesis of Indolizidine 167B both in racemic and optically active form [7–8]; the crucial key was the cyclodehydration of 4-carboxyethyl-4-(pyrrol-1-yl)butanal, obtained *via* selective reduction of pyrrole masked glutamic acid diethyl ester hydrochloride [9], to the corresponding 5,6-dihydroindolizine bearing the carboxyethyl group in position five. Successively a series of ester group transformations to *n*-propyl group followed by final exhaustive hydrogenation gave the desired product. In the synthesis of Indolizidine 167B depicted here the construction of the bicyclic core still occurs via a pyrrolylbutanal; unlike the previous case, the aldehyde comes from rhodium-catalyzed hydroformylation of optically active (*R*)-3-(pyrrol-1-yl)hex-1-ene (**1**) (Scheme 1). The formed linear aldehyde **2a** (Scheme 2), bearing an *n*-propyl group in the required position, undergoes sequential intramolecular cyclization/dehydration/hydrogenation *in situ* to give 5-*n*-propyl-5,6,7,8-tetrahydroindolizine (**4**), *via* 5-*n*-propyl-5,6-dihydroindolizine (**3**) having the same optical purity as the starting olefin; a successive enantioselective reduction gives the target compound (ee 92%) (Scheme 1).

The rhodium-catalyzed hydroformylation of olefins is an important industrial tool for the production of aldehydes [10–11]. During the last few years, the *oxo* process has been employed also in the synthesis of fine chemicals especially integrated in multi-step domino reaction sequences which are a very convenient approach to complex architectures in one simple, safe, environmentally acceptable and resource-effective operation [12–14]. The mechanistic as well as synthetic implications of the *oxo* reaction involving vinyl and allyl aromatic and heteroaromatic olefins has a topic of our research for many years [15–19]; now it is the first time that the rhodium catalyzed hydroformylation is employed by us in the total synthesis of a target compound and as a key reaction in a domino process with a high number of steps.

## Results and Discussion

The optically active starting material **1** (ee 92%) was prepared from the corresponding amino acid D-norvaline as previously reported [20]. Then **1** was introduced in a 25 ml stainless steel autoclave, in the presence of Rh_4_(CO)_12_ as a catalyst precursor (Rh/substrate = 1/100), at 125 °C and 30 atm total pressure (CO/H_2_ = 1:1), in toluene as solvent. After 25 min, the olefin was completely absent and 5-*n*-propyl-5,6-dihydroindolizine (**3**) was the predominant product (Scheme 1). As far as the typical *oxo* products are concerned, i. e. the aldehyde isomers, the linear **2a** was present only in trace amounts in the reaction mixture while the branched one **2b** was in 13% (GC-MS control) with respect to the indolizine structure (Scheme 2). While at room temperature and high pressure the **2a**/**2b** ratio is largely favorable to the branched aldehyde (29/71) [21], under the above conditions (high temperature and low pressure) a highly regioselective hydroformylation into the linear aldehyde takes place; this is a consequence of the isomerization of the branched alkyl-rhodium intermediate **b**, precursor of **2b**, into the linear one **l**, precursor of **2a**, *via* a ß-elimination process with formation of olefin **1** (Scheme 2) [22]. This transformation is completely stereospecific and it does not involve the chiral center. An evaluation of the enantiomeric excess of both unconverted **1** and produced **3** was carried out in order to test the configurational stability of these structures under hydroformylation conditions. Interestingly **1** showed, at all conversions, practically the same ee, that is, the starting ee value (92%). A similar behaviour occurred for dihydroindolizine **3**, its ee value remaining the same as that of the corresponding olefin **1** (ee 92%) at all reaction times. The isomerization of **b** into **l** and the absence of racemization of starting substrate are the peculiar features of this process. Indeed, under the adopted experimental conditions, aldehyde **2a**, as it forms, reacts further in an *in situ* intramolecular electrophilic substitution on position two of the pyrrole nucleus giving dihydroindolizine **3**, likely *via* formation of the bicyclic alcohol **2'a** followed by dehydration (Scheme 2). Thus the cyclization of the linear aldehyde results much faster than hydroformylation while the same reaction does not occur for the branched one, which remains unaltered in the reaction mixture.

Compound **3** is stable enough to be handled easily at room temperature without any decomposition or change of enantiomeric excess. When, at complete conversion of **1**, the gas mixture was removed from the crude hydroformylation product and H_2_ (50 atm) was added and the reaction vessel heated for a long time (12 h), **3** disappeared and the corresponding 5,6,7,8-tetrahydroindolizine **4** was obtained (Scheme 1; ii), together with the diastereoisomeric alcohols coming from the branched aldehyde: additional reduction of the pyrrole nucleus was never observed even by forcing the conditions (high pressure and high temperature). This goal was successfully reached by treating **4** (or **3**) with Rh on carbon 5% as catalyst precursor, under H_2_ (10 atm), with the hydrogenation time varying from 60 min to 45 min respectively. In both cases only the diastereomer corresponding to indolizidine 167B, characterized by C5 and C9 chiral centers with the same absolute configuration, was obtained, the reaction being completely stereoselective as evidenced by comparison with literature data for the same isomer [23]. It is to remarkable that the global synthesis is completely stereospecific, with the final product having the same optical purity as the starting olefin (ee 92%).

## Conclusions

We describe here a new synthesis of optically active (–)-Indolizidine 167B based on regioselective hydroformylation/intramolecular cyclization reactions which provides a general approach to the indolizine nucleus. It is a multi-step domino process which starts with the interconversion of the isomeric rhodium-alkyl intermediates and carries on with the intramolecular cyclodehydration of the formed linear aldehyde followed by hydrogenation. All steps occur with almost complete configurational stability and the final indolizine has the same optical purity as the starting material. The hydroformylation conditions are perfectly compatible with optically active pyrrolylolefins, and the *oxo* process is proposed as a convenient instrument for indolizine synthesis in general.

## Supporting Information

File 1 Experimental data. This file contains all experimental methods and analytical data belonging to the compounds described in the article.
