# Supplementary material for: Synthesis of (–)-Indolizidine 167B based on domino hydroformylation/cyclization reactions
Source: Beilstein J Org Chem. 2008 Jan 15;4:2. doi: 10.1186/1860-5397-4-2 (PMC2241606; doi:10.1186/1860-5397-4-2)
Supplement: File 1 — Experimental data. This file contains all experimental methods and analytical data belonging to the compounds described in the article. [file Beilstein_J_Org_Chem-04-02-s001.doc]

**Experimental data**

All reagents were purchased in the highest purification grade available. TLC analyses were performed on aluminum oxide 60 F254 neutral plats from Merck. For preparative chromatography Merck aluminum oxide 90 active (neutral, 70-230 mesh) was used. Toluene was dried over molecular sieves and distilled under nitrogen. Gas-chromatographic analysis were performed on a Perkin Elmer 8700 chromatograph equipped with a DB1 15 m x 0.25 mm capillary column, using nitrogen as carrier gas. GC/MS analyses for all the other derivatives were performed on a Perkin Elmer Q-Mass 910, interfaced with a Perkin Elmer 8500 chromatograph equipped with a 30 m x 0.25 mm apolar DB1 capillary column, using helium as carrier gas. The determination of the ee was carried out by gas chromatography with the chiral capillary column CHIRALDEX G-TA (γ-cyclodextrin trifluoroacetyl, 50 mx 0.25mm). Optical rotations were measured with a JASCO DIP-370 digital polarimeter at the given temperature. ^1^H NMR spectra were recorded at 200 MHz and ^13^C NMR spectra were recorded at 50 MHz, on a Varian Gemini 200 in CDCl_3_. Chemical shifts are reported in δ (ppm) relative to CDCl_3_ and signals are indicated as s (singlet), bs (broad singlet), d (doublet), dd (double doublet), t (triplet), q (quartet), m (multiplet). (*R*)-3-(pyrrol-1-yl)hex-1-ene (**1)** was synthesized following a known procedure.[20] Rh_4_(CO)_12_ was prepared as reported in literature.[24-25]

Hydroformylation of (*R*)-3-(pyrrol-1-yl)hex-1-ene (**1)**: preparation of (-)-(*R*)-5-n-propyl-5,6-dihydroindolizine **3**. A solution of **1** (3.36 mmol) and Rh_4_(CO)_12_ (substrate/Rh=100/1) in toluene (10 ml) was introduced by suction into an evacuated 25 ml stainless steel reaction vessel. Carbon monoxide was introduced, the autoclave was then rocked, heated to the reaction temperature and hydrogen was rapidly introduced to give the desired total pressure (CO/H_2_=1/1). When the gas absorption reached the value corresponding to the desired conversion, the reaction vessel was rapidly cooled, and the reaction mixture was siphoned out and GC/GC-MS was used to determine the composition of the reaction mixture. Then the solvent was evaporated under reduced pressure and the residue was eluted on chromatographic column (CH_2_Cl_2_/hexane=1/7) giving (-)-(*R*)-5-n-propyl-5,6-dihydroindolizine **3** as an orange oil (76% yield). Selected data for **3**: [α]_D_^26°^ = -43.5 (CH_2_Cl_2_, c=0.86). ^1^H NMR δ 6.68 (t, J=1.8 Hz, 1H, H3), 6.42 (d, J=9.8 Hz, 1H, H8), 6.13 (t, J=3.1 Hz, 1H, H2), 6.03 (bs, 1H, H1), 5.63 (m, 1H, H7), 4.04 (q, J=6.5 Hz, 1H, H5), 2.68 (m, 1H, H6), 2.28 (m, 1H, H6'), 1.85-1.25 (m, 4H, CH_2_-CH_2_), 0.95 (t, J=7.5 Hz, 3H, CH_3_). ^13^C NMR δ 13.6, 18.9, 28.9, 36.2, 53.8, 105.6, 107.1, 117.2, 119.3, 119.6, 127.9. MS m/e 161 (M^+^ 40), 132 (6), 118 (100), 91 (8).

Selected data for 5,6,7,8-tetrahydroindolizine **4**. As a colorless oil after elution on chromatographic column (hexane). ^1^H NMR δ 6.65 (t, J= 1.4 Hz, 1H, H3), 6.12 (t, J= 2.0 Hz, 1H, H2), 5.81 (bs, 1H, H1), 3.96 (m, 1H, H5), 2.75 (m, 2H, H8), 2.03 (m, 2H, CH_2_), 1.91 (m, 2H, CH_2_), 1.68 (m, 2H, CH_2_), 1.41 (m, 2H, CH_2_), 0.97 (t, J= 4.8 Hz, 3H, CH_3_). MS m/e 163 (M^+^ 57), 121 (100), 120 (91), 106 (17), 93 (21), 80 (19).

(-)-Indolizidine 167B.

Step iii and iv. A solution of **3** or **4** ( 80 mg ) in Et_2_O ( 5 ml) containing 5% Rh-C ( 85 mg) was submitted under H_2_ 10 atm and vigorous stirring in a stainless steel vessel at room temperature for 60-45 min. The catalyst was removed by filtration and the solution concentrated in vacuo to afford (-)-Indolizidine 167B as a colorless oil. The spectroscopic data of this product are in complete agreement with the reported data:[23] [α]_D_^26°^ = -81.3 (CH_2_Cl_2_, c=0.95).^1^H NMR δ 3.23 (td, J=9.1 Hz, 1H, H3_eq_), 1.91 (q, J=9.1 Hz, 1H, H3_ax_), 1.80-1.10 (m, 16H), 0.87 (t, J=7.1 Hz, 3H, CH_3_). ^13^C NMR δ 65.2, 63.5, 51.3, 37.1, 31.1, 30.9, 30.5, 24.7, 20.8, 19.1, 14.8.
